# Role of Cytokines in Wound Healing Following Wound Catheter Analgesia in Rats

**DOI:** 10.3390/vetsci12121214

**Published:** 2025-12-18

**Authors:** Marija Lipar, Andrea Martinović, Tamara Nikuševa Martić, Tihana Kurtović, Jadranka Bubić Špoljar, Andrea Gelemanović, Marko Hohšteter, Lidija Medven Zagradišnik, Ivana Mihoković Buhin, Andrija Musulin, Višnja Nesek Adam, Božo Gorjanc, Slobodan Vukičević, Dražen Vnuk

**Affiliations:** 1 Clinic for Surgery, Orthopaedics and Ophthalmology, Faculty of Veterinary Medicine, University of Zagreb, 10000 Zagreb, Croatia; mlipar@vef.unizg.hr (M.L.); dvnuk@vef.unizg.hr (D.V.); 2 Faculty of Veterinary Medicine, University of Zagreb, 10000 Zagreb, Croatia; andrea-1510@live.com; 3 Department of Biology, School of Medicine, University of Zagreb, 10000 Zagreb, Croatia; tamara.nikuseva.martic@mef.hr (T.N.M.); jadranka.bubic@mef.hr (J.B.Š.); 4 Centre for Research and Knowledge Transfer in Biotechnology, University of Zagreb, 10000 Zagreb, Croatia; tihana.kurtovic@unizg.hr; 5 Mediterranean Institute for Life Sciences (MedILS), University of Split, 21000 Split, Croatia; agelemanovic@medils.unist.hr; 6 Bioinstitut Ltd., 40000 Čakovec, Croatia; marko.hohsteter@bioinstitut.hr; 7 Department of Veterinary Pathology, Faculty of Veterinary Medicine, 10000 Zagreb, Croatia; imihokovic@vef.unizg.hr; 8 Klinika Buba—Vetti Group, 10000 Zagreb, Croatia; andrijamusulin@gmail.com; 9 General Hospital Sveti Duh, 10000 Zagreb, Croatia; visnja.nesek@hotmail.com; 10 Department of Plastic and Reconstructive Surgery, School of Medicine, 10000 Zagreb, Croatia; gorjancb@yahoo.com; 11 Laboratory for Mineralized Tissues, School of Medicine, 10000 Zagreb, Croatia; slobodan.vukicevic@mef.hr

**Keywords:** angiogenesis, apoptosis, buprenorphine, levobupivacaine, meloxicam, TGF-β1, wound healing

## Abstract

Background: Local analgesia administered through a wound catheter is widely used for postoperative pain control, yet its effects on wound healing remain incompletely understood. This study examined how levobupivacaine alone or combined with meloxicam or buprenorphine influences inflammatory markers, angiogenesis, apoptosis, and transforming growth factor β1 (TGF-β1) expression during wound healing in rats. Methods: Thirty Sprague Dawley rats were assigned to five groups: control, saline, levobupivacaine (L), levobupivacaine/meloxicam (L/MEL), and levobupivacaine/buprenorphine (L/BUP). Treatments were administered via a wound catheter for three days. Blood and skin samples were collected before surgery and on days 3, 10, and 21. Results: Levobupivacaine combined with meloxicam or buprenorphine caused fluctuations in white blood cell counts, while albumin levels remained stable. Angiogenesis in the L/MEL group was markedly increased compared with the control, saline, and levobupivacaine-only groups, but the newly formed vessels exhibited consistently narrow lumina during the early healing phase. Caspase-3–positive cells were most numerous in L/MEL during inflammatory and proliferative phases, whereas delayed caspase-3 activation was observed in L/BUP. TGF-β1 expression peaked in both adjuvant groups on days 3 and 10. Conclusions: Meloxicam and buprenorphine increased TGF-β1 expression, but their vascular effects differed considerably. Meloxicam induced a marked increase in angiogenesis, but the newly formed vessels were structurally immature, displaying uniformly narrow lumina and poor architectural organisation, which led to their subsequent regression. In contrast, buprenorphine supported the formation of more mature vascular structures, characterised by wider vessel lumina and a more organised vascular network. These findings demonstrate that adjuvants used with levobupivacaine can significantly modify angiogenic and apoptotic responses and should be carefully considered when selecting multimodal local analgesia strategies after surgery.

## Simple Summary

Effective postoperative pain control is important for proper wound healing, but analgesic drugs may also influence biological processes involved in tissue repair. In this study, we examined how levobupivacaine, a commonly used local anaesthetic, affects wound healing when delivered through a wound catheter, either alone or in combination with meloxicam or buprenorphine. Rats received these treatments for the first three days after surgery, and blood and skin samples were analysed on days 3, 10, and 21. We focused on inflammation, new blood vessel formation, apoptosis, and the expression of transforming growth factor β1 (TGF-β1), a key regulator of healing. Meloxicam combined with levobupivacaine caused markedly increased angiogenesis compared with levobupivacaine alone, characterised by a high number of vessels with very narrow lumina early after surgery. It also produced a higher number of apoptotic cells than both the control and levobupivacaine groups during the early healing phase. Buprenorphine showed delayed activation of apoptosis relative to levobupivacaine alone and supported angiogenesis, characterised by vessels with wider lumina compared with the meloxicam group. Both meloxicam and buprenorphine increased TGF-β1 expression, especially on days 3 and 10. These findings suggest that adjuvants used with local anaesthetics can significantly alter healing processes. Therefore, the choice of analgesic combination should be based on a consideration not only of pain control but also of its potential impact on wound healing.

## 1. Introduction

Postoperative pain management is an essential component of anaesthesia, as it minimises complications and facilitates recovery. Numerous local and regional analgesic techniques have been described, but many are time-consuming, expensive, or associated with a relatively high complication rate. The use of a wound infiltration catheter represents a simple and rapid technique that prolongs analgesia by delivering long-acting local anaesthetics such as bupivacaine and levobupivacaine. Adjuvants added to local anaesthetics may enhance or prolong their effects. Local anaesthetics act by blocking sodium channels on nerve fibres and γ-aminobutyric acid (GABA) neurotransmission, thereby preventing the transmission of nociceptive signals from the wound surface [1]. Their distribution depends on lipid solubility, protein binding, and regional blood flow [2]. Levobupivacaine is a long-acting amide anaesthetic with a favourable safety profile, which has contributed to its widespread clinical use [3].

Meloxicam is a selective cyclooxygenase-2 (COX-2) inhibitor recommended for various pain conditions, with adverse effects comparable to other NSAIDs [4]. Buprenorphine is a potent opioid analgesic with slow receptor dissociation and prolonged action [5]. A potential disadvantage of wound catheter use is an increased risk of infection due to bacterial migration along the catheter, as well as possible effects of the catheter and the administered drugs on wound healing [1]. Levobupivacaine has been shown to increase inflammatory activity compared with untreated incisional wounds, with a dose-dependent enhancement of inflammation lasting up to eight days after injury [6], and may negatively affect later stages of wound healing [7]. Wound healing comprises four overlapping phases: haemostasis, inflammation, proliferation, and remodelling. Tissue repair begins during inflammation and depends on the activity of immune cells, particularly M1 and M2 macrophages. M1 macrophages produce pro-inflammatory cytokines such as interleukin-6 (IL-6), whereas M2 macrophages produce anti-inflammatory cytokines, including transforming growth factor β1 (TGF-β1), which promotes tissue repair. Injured tissues produce exudate containing white blood cells, C-reactive protein (CRP), and other serum proteins [8,9]. CRP is an acute-phase protein (APP), while albumin is a negative-phase protein (NPP), with most albumin distributed in interstitial spaces where it contributes to osmotic pressure and acts as a predominant antioxidant. APPs peak within 24–48 h after injury, whereas NPPs increase later [10]. TGF-β1 regulates cell proliferation, differentiation, migration, and apoptosis [11,12,13,14]. Apoptosis, or programmed cell death, maintains tissue homeostasis and is characterised by cell shrinkage, membrane blebbing, chromatin condensation, and DNA fragmentation [15].

Revascularisation and inflammatory regulation represent the most vulnerable stages of healing. Cluster of differentiation 31 (CD31), also known as platelet endothelial cell adhesion molecule-1 (PECAM-1), is highly expressed on endothelial cells and participates in leukocyte trans-endothelial migration during homeostasis and inflammation [16,17,18].

The main aim of this study was to elucidate the effects of locally applied levobupivacaine, alone or combined with meloxicam or buprenorphine, administered through a wound catheter as part of a multimodal pain management approach, on surgical wound healing in rats.

## 2. Materials and Methods

### 2.1. Animals

Study protocols were conducted on male and female Sprague Dawley rats (Rattus norvegicus), aged six months and weighing 150–200 g. Animals were randomly allocated to five groups of six rats each. The study was approved by the Ministry of Agriculture (permit no. 525-10/0543-20-4). Rats were housed individually under standard laboratory conditions with environmental enrichment, ad libitum access to water, and a standard diet. All procedures complied with institutional guidelines, the 3R principles, and EU Directive 2010/63. Animals were monitored post-procedurally for gait abnormalities, ocular and mucosal changes, head posture, respiratory patterns, hydration status, appetite, body condition, and behavioural deviations.

### 2.2. Experimental Design

Anaesthesia was induced using a combination of dexmedetomidine (Sedadex, Dechra, Northwich, UK) at 0.5 mg/kg, ketamine (Ketamin, Abbott, Maidenhead, UK) at 75 mg/kg, and butorphanol (Torphadine 10 mg/mL, Dechra, UK) at 1 mg/kg, administered intramuscularly. The dorsal thoracic area (approximately 5 × 10 cm) was prepared aseptically and draped. A skin incision was made in the left paramedian line (0.5 cm lateral and parallel to the dorsal midline) with a total length of 4 cm.

#### Grouping

In Group 1, the skin incision was closed with 4-0 nylon (Dermalon™, Covidien, Dublin, Ireland) using a simple interrupted suture pattern, without placement of a wound catheter (control group).

In the remaining four experimental groups, a wound catheter was inserted beneath the skin incision. Cranially to the primary incision, the catheter was exteriorised through a newly created skin opening and secured using a Chinese finger-trap friction suture with 4-0 nylon. The wound was then sutured over the catheter.

In Group 2, sterile saline (0.9% NaCl, Braun, Waiblingen, Germany) was administered through the catheter every 24 h for three consecutive days.

In Group 3, levobupivacaine (2.5 mg/kg; Levobupivakain Kabi 5 mg/mL, Fresenius, Bad Homburg, Germany) (L group) was administered.

In Group 4, a combination of levobupivacaine (2.5 mg/kg) and meloxicam (1 mg/kg; Meloxidolor, Genera, Zagreb, Croatia) (L/MEL group) was administered.

In Group 5, a combination of levobupivacaine (2.5 mg/kg) and buprenorphine (0.02 mg/kg; Bupredine multidose 0.3 mg/mL, Dechra, UK) (L/BUP group) was administered.

All medications were diluted with saline in the syringe to a final volume of 2 mL and delivered through the catheter once daily during the first three postoperative days. The wound catheter was removed on postoperative day 3.

No prophylactic or therapeutic antibiotics were administered locally or systemically.

### 2.3. Skin Sampling

Rats were anaesthetised to obtain skin samples for histological and immunohistochemical assessment at the following time points: prior to surgery and on days 3, 10, and 21 post-surgery. Anaesthesia was the same as in the initial surgical procedure. Skin biopsies were taken from the incisional wound using a 6 mm punch biopsy tool. Samples on days 3, 10, and 21 were collected in a cranial-to-caudal direction. Punch biopsy wounds were closed using two skin staples. Euthanasia was performed on day 21 post-surgery under general anaesthesia (as mentioned above) using T-61 (Euthasol^®^, Genera, Croatia) administered intrapulmonarily.

### 2.4. Blood Sampling and Analyses

Under general anaesthesia at each time point, blood samples were collected from the orbital sinus into sterile tubes for haematological and biochemical analysis. Blood sampling was performed immediately prior to each skin biopsy (zero time point) and on days 3 (time point 3), 10 (time point 10). and 21 (time point 21) post-surgery. Following sampling, serum was separated and stored at −80 °C until analysis.

#### 2.4.1. Albumin and Complete Blood Count

Albumin concentrations were measured using an absorption spectrophotometry analyser (VetTest, Idexx, Westbrook, MA, USA). Complete blood cell counts were obtained using an automated haematology analyser within 2 h of sample collection.

#### 2.4.2. ELISA Tests

Levels of TGF-β1 and CRP in rat serum were determined as follows. TGF-β1 was measured using the TGF beta 1 Rat ELISA kit (Abcam, Cambridge, UK; ab119558, UK). Each sample was prepared at a 250-fold dilution. CRP was measured using the Rat CRP/C-Reactive Protein ELISA kit (Sigma-Aldrich, Kent, UK, RAB0097, UK), with each sample diluted 50,000-fold. All samples were analysed in duplicate. ELISA assays were performed according to the manufacturer’s instructions.

### 2.5. Histological Analysis

Skin samples were fixed in 4% buffered formalin for 24 h, paraffin-embedded, and cut into 4 μm thick histological sections. Samples were stained with haematoxylin–eosin and covered with a coverslip. Histopathological slides were analysed using a Nikon 2000 Eclipse light microscope (Nikon Corporation, Tokyo, Japan). Representative wound areas were photographed with an Olympus DP20 camera (part of the Nikon 2000 Eclipse microscope system equipped with Cell B–Olympus software) using 4× and 10× objective lenses.

The inflammatory reaction and the amount of granulation tissue were assessed using pre-established criteria: 0 (none), 1 (mild), 2 (moderate), and 3 (severe) [19,20]. Collagen fibre orientation was evaluated as 1 (vertical), 2 (mixed), or 3 (horizontal), and the collagen pattern was graded as 1 (reticular), 2 (mixed), or 3 (fascicle) [20].

The following morphometric characteristics were measured for each sample according to Lemo et al. [21]: length of the re-epithelialisation zone (Lr), distance between the borders (S), depth of the wound (D), thickness of the connective tissue in the wound centre (T), and thickness of the natural dermis on both sides of the wound (N).

### 2.6. Immunohistochemical Analysis

#### 2.6.1. Primary Antibodies

Immunohistochemistry (IHC) was performed using two primary antibodies: anti-CD31 (Monoclonal Mouse Anti-Human CD31, Clone JC70A, Dako, Glostrup, Denmark) diluted 1:20, and anti-caspase-3 (Polyclonal Rabbit Anti-Caspase-3, active/cleaved form, Merck Millipore, Burlington, MA, USA) diluted 1:100.

#### 2.6.2. IHC Procedure

Tissue sections were deparaffinised, rehydrated, and cut to a thickness of 4 μm. Antigen retrieval was performed by heat treatment in target retrieval solution, pH 6.0 (Dako REAL™ Target Retrieval Solution, S2031, Dako Denmark A/S, Glostrup, Denmark), for 20 min. EnVision Peroxidase-Blocking Reagent (Dako, Glostrup, Denmark) was applied for 5 min for both antibodies.

An additional blocking step using 5% bovine serum albumin (BSA, Sigma-Aldrich) in phosphate-buffered saline (PBS) was applied for 30 min prior to incubation with the anti-caspase-3 antibody.

Incubation with the anti-CD31 antibody was performed at room temperature for 30 min, whereas incubation with the anti-caspase-3 antibody was performed overnight at 4 °C.

The Dako REAL™ EnVision™ Detection System, Peroxidase-DAB, Rabbit/Mouse (K5007), was used for visualisation of both antibodies. IHC staining was performed automatically using the Dako Autostainer Plus (Fort Collins, CO, USA). Kidney tissue and small intestine tissue served as positive controls for anti-CD31 and anti-caspase-3, respectively.

CD31 was used to visualise blood vessels. The following parameters were derived from CD31 staining: number of vessels, average vessel perimeter, and total vessel area.

#### 2.6.3. Immunohistochemical Scoring

IHC slides were evaluated independently by three pathologists, and IHC positivity was assessed using 10× and 40× objective lenses. For each CD31-stained sample, the total wound area (μm^2^), number of vessels, total and average vessel area (μm^2^), average vessel perimeter (μm), and vessel-area-to-total-area percentage were quantified using Cell B–Olympus software [22].

Immunoreactivity of caspase-3 was assessed by estimating the number of positive signals within the total wound area (No/μm^2^) [23].

### 2.7. Statistical Analysis

The zero time-point sample was obtained from one randomly selected rat from each group.

Data are presented as mean ± standard deviation (SD). To evaluate statistical differences and to examine the effects of time, group, and their interaction, one-way and two-way ANOVA were performed, followed by Tukey’s HSD post hoc tests with Benjamini–Hochberg *p*-value adjustment to control for multiple hypothesis testing. Pearson correlation was used to assess associations between selected variable pairs.

The significance level was set at *p* < 0.05 for all analyses. Statistical analyses were performed using the R statistical computing environment (version 4.0.0). Correlation figures were generated using the ggplot2 package (version 3.3.5) [24,25].

Supporting data and materials are provided in the Supplementary Materials File.

## 3. Results

### 3.1. Differences Between Groups

Wound healing was monitored for 21 days following the surgical procedure and topical treatment of the wound with levobupivacaine alone or in combination with the opioid or NSAID adjuvants.

**Figure 1:**
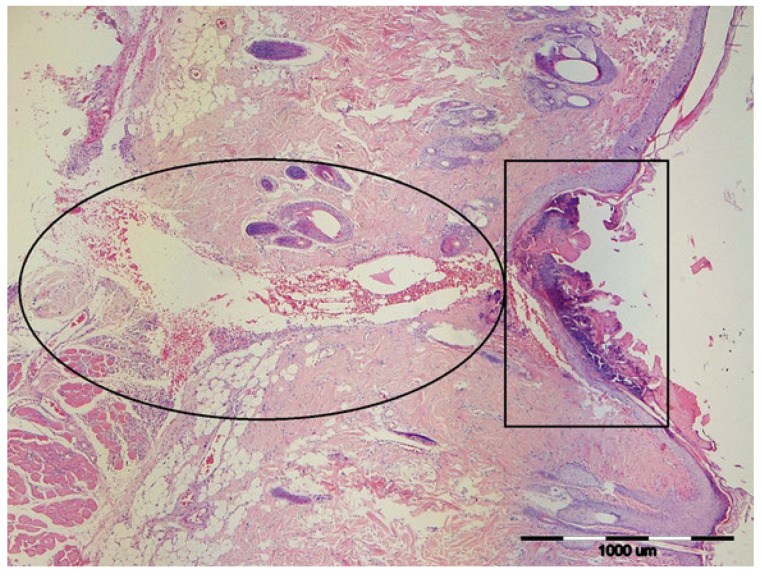
Representative histological image of the wound area from Group 2, Day 3. The image shows the epidermis, dermis, and subcutis with a surface crust (rectangle) and haemorrhage in the dermis and subcutis (ellipse). Rat, skin, dermis. H&E, 40×.

As shown in Figure 1, the wound on Day 3 exhibited typical early inflammatory morphology, including a surface crust and dermal/subcutaneous haemorrhage.

In Group 3, no significant differences were observed in TGF-β1 concentrations across time points, whereas in the remaining four groups, considerable differences were detected both within the same group at different time points and between groups at identical time points.

C-reactive protein (CRP) in Group 1 (control) increased steadily during the study and differed significantly between time points. In Group 2, a CRP peak was observed on day 10, representing the highest overall measured value. In Group 3 (levobupivacaine), no significant differences were observed between time points. Compared with the control group, all groups showed elevated CRP concentrations at every postoperative time point, except Group 2 on day 21 and Group 3 on day 10. A significant difference was noted between Groups 1 and 5 on day 3.

White blood cell (WBC) count differed significantly only between time points 3 and 21 in Groups 4 and 5, although all measured values remained within physiological ranges. Groups 4 and 5 exhibited the most pronounced fluctuations in WBC count. At the final time point, significant differences were observed between Groups 1 and 4, 1 and 5, 2 and 4, and 2 and 5, with Groups 4 and 5 showing significantly lower WBC values. Table 1 presents the comparison of TGF-β1, CRP, and WBC levels within the five groups across the four time points.

Group 3 exhibited the greatest variability in the length of the re-epithelialisation zone (L). In Groups 4 and 5 on day 21, the re-epithelialisation zone was smaller compared to other groups, although this difference was not significant. Only Group 2 showed no significant differences across time points.

**Figure 2:**
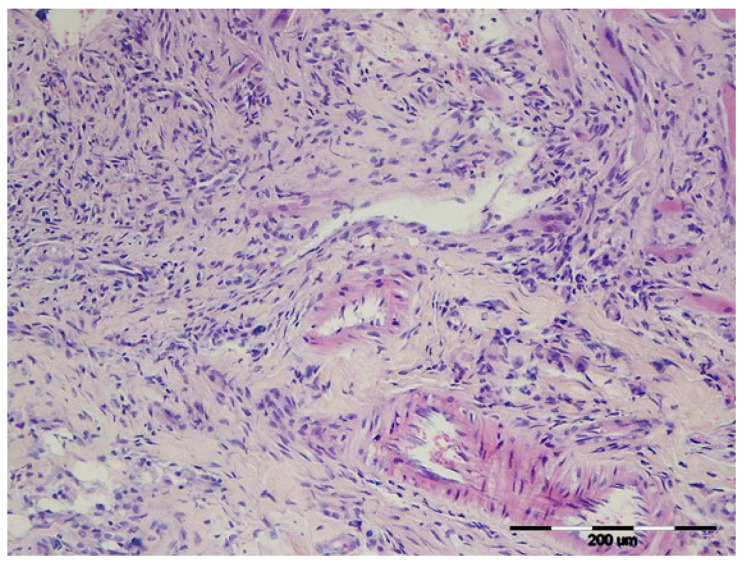
Rat, skin, dermis. Representative histological image of granulation tissue from the Group 3 on Day 10, showing abundant proliferative fibroblasts, collagen fibres, and neovascularisation typical of the proliferative phase. Rat, skin, dermis. H&E, 200×.

Thickness of the connective tissue (T) was greatest in Group 3 on day 10 (Figure 2), with similar results in Group 4.

In the L/MEL group, the increased connective-tissue thickness did not correspond to enhanced collagen deposition. Histologically, this group showed oedematous and disorganised granulation tissue with loosely arranged and partially fragmented collagen fibres, together with abundant inflammatory and apoptotic cells, indicating delayed matrix maturation. These two groups also showed the largest differences between time points within the same group. In Groups 1, 2, and 5, no significant differences were observed between time points.

Significant changes in the number of vessels were noted in Groups 3 and 4 at different time points, as well as on day 21 between Groups 1 and 2 and between Groups 1 and 4. On day 10, the highest vessel counts were observed across all groups.

The most notable changes in average vessel perimeter occurred in Group 4. In Groups 2, 3, and 4, significant changes were observed during the inflammatory and proliferative phases of healing.

**Figure 3:**
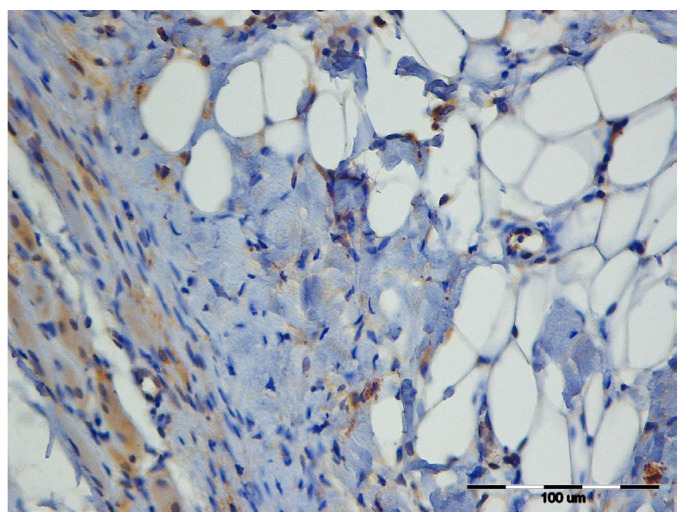
Representative image of caspase-3 activity in the wound area from Group 3 on Day 3, showing numerous caspase-3–positive cells (brown staining). Rat, skin, dermis. IHC, caspase-3, 400×.

Group 3 showed significant differences in the average number of caspase-3–positive cells across time points. Significant intergroup differences were also observed during the proliferative phase. Figure 3 illustrates these findings.

Table 2 compares the length of the re-epithelialisation zone (L), connective tissue thickness (T), number and average perimeter of vessels, and the number of caspase-3–positive cells across the five groups at all four time points.

Albumin concentrations remained within physiological ranges and did not differ significantly between groups or time points.

Red blood cell (RBC) count decreased from the zero time point to day 3 in all groups, followed by a gradual increase until days 10 and 21, when values approached baseline in all groups. Significant differences between time points were observed in all groups except Group 4. No significant differences were observed between groups at the same time point. Albumin and RBC results are presented in Supplementary Table S1.

For the distance between wound borders (S), no significant changes across time points were detected in Group 2. The S parameter was nearly identical in Groups 3 and 4 on day 21. In Group 3, significant differences were observed on days 10 and 21 compared with the zero time point, whereas in Group 4, significant differences were detected between all time points.

Wound depth (D) differed significantly across time points only in Group 5. On day 3, Group 5 exhibited the greatest wound depth. No other significant differences were recorded. These differences in wound depth (D) are presented in Supplementary Table S2.

Thickness of the natural dermis (N) changed significantly across time points in Groups 1, 2, and 3, with no significant changes in Groups 4 and 5. Values for S, D, and N are presented in Supplementary Table S2.

Significant changes in total wound area were observed on days 3, 10, and 21 in Group 3, and on days 3 and 21 in Group 4. A significant difference was found between Groups 1 and 4 on day 21. There were no significant changes in total vessel area between groups or across time points.

**Figure 4:**
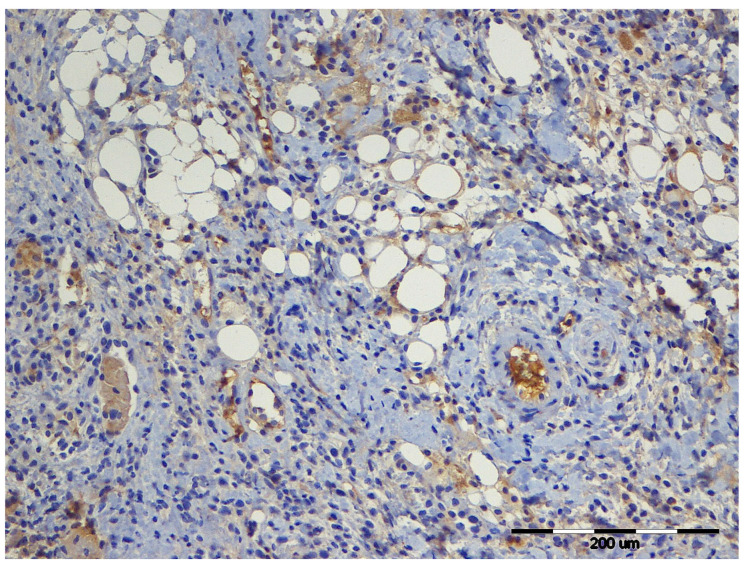
CD31 immunohistochemistry of the wound area from Group 5 on Day 21, showing abundant wide-lumen mature vessels characteristic of late-stage angiogenesis. Rat, skin, dermis. IHC, CD31, 200×.

Average vessel area differed significantly in Groups 2 and 4, with Group 4 showing the lowest value on day 3. To illustrate the morphological appearance of late-stage angiogenesis, Figure 4 presents a representative CD31-stained section from Group 5 (levobupivacaine/buprenorphine) on Day 21. At this time point, quantitative analysis showed that this group exhibited the highest vessel-area percentage among all groups.

Average vessel perimeter and percentage of vessel area were most affected in the NSAID-containing group and were not influenced by opioid treatment.

Group 4 showed the highest number of caspase-3–positive cells on days 3 and 10 and the lowest values on day 21. Results are presented in Supplementary Table S3.

### 3.2. Correlation Results

CRP and albumin showed a significant negative correlation on day 3 in Groups 3 and 4.

CRP and WBC were negatively correlated in Group 3 on day 3, but positively correlated on day 10. A positive correlation between CRP and WBC was also observed in Group 4 on day 21.

The number of vessels and the average number of caspase-3–positive cells showed a significant positive correlation in Groups 2 and 5 on day 21.

## 4. Discussion

Wound healing is a complex and dynamic overlapping multistep process composed of four basic stages: haemostasis (0–several hours after injury), inflammation (1–3 days), proliferation (4–21 days), and remodelling (21 days–1 year) [8,9]. In this study, skin and blood samples were collected on days 3, 10, and 21 following surgery. Ineffective post-surgical analgesia can delay wound healing [26,27]. Therefore, we investigated the effect of locally applied levobupivacaine, alone or combined with meloxicam or buprenorphine, on wound healing. The results indicate that meloxicam stimulates TGF-β1 production in all phases of healing and induces inflammation and angiogenesis with small-lumen vessels in the early phase, while also increasing caspase-3 activity. Buprenorphine provoked angiogenesis with wide-lumen vessels in the initial stages, likely via opioid receptor stimulation, and inhibited caspase-3 activity during the inflammatory phase.

During inflammation, tissue injury induces elevated IL-6 production, which stimulates hepatic synthesis of acute phase proteins (APPs) such as CRP, serum amyloid A, fibrinogen, haptoglobin, and α1-antichymotrypsin [28]. This is consistent with our findings, where significant CRP differences were recorded not only during inflammation but also in the remodelling phase in all treated groups except the levobupivacaine group. Protein catabolism was not detected, as albumin levels differed only minimally from baseline, suggesting that albumin does not influence wound healing in this model. The negative correlation between CRP and WBC likely reflects strong CRP affinity for microbial and damaged-cell membrane lipids, enhancing opsonisation and phagocytosis by WBCs during inflammation [29]. Caspase-3-positive cells were also elevated, confirming the progression of apoptosis.

IL-6 decreases the production of fibronectin, albumin, and transferrin, which explains reduced serum albumin during acute inflammation. It also stimulates macrophage maturation in the bone marrow, resulting in increased platelet and RBC release into circulation [28]. In contrast to RBCs, albumin concentrations did not significantly differ between groups. WBCs differed significantly in the L/MEL and L/BUP groups, where TGF-β1 signalling likely promotes alternative macrophage activation [11]. TGF-β1 production, probably originating from platelets, was stimulated by NSAID treatment during the initial inflammatory phase. Neither the incision alone nor the catheter placement with levobupivacaine induced sufficient bone marrow stimulation to alter WBC or RBC production.

Tanaka et al. demonstrated a negative correlation between IL-6 and TGF-β1, as IL-6 inhibits TGF-β1 production [28]. This suggests that Groups 4 (L/MEL) and 5 (L/BUP) likely had lower IL-6 levels, prolonging inflammation—a finding supported by CRP dynamics.

TGF-β1 is a multifunctional cytokine acting as a molecular sensor of tissue injury. It binds to serine/threonine kinase receptors and triggers alternative macrophage activation [11,30], exists as a latent complex in the ECM, and functions as a promigratory factor for adult stem-cell mobilisation during repair [31]. TGF-β (predominantly TGF-β1) is embedded in ECM-stimulated WBC/macrophage production until day 10 post-injury [32]. In this study, NSAIDs and opioids stimulated WBC production more strongly than local anaesthetic alone. Saline flushing likely disrupted coagulum formation and reduced TGF-β1 expression compared with groups receiving levobupivacaine alone (Group 3) or in combination with meloxicam (Group 4) or buprenorphine (Group 5). Thrombospondin-1, involved in dermal fibrosis and TGF-β activation, may prolong inflammation, angiogenesis, and neovascularisation [31]. In the remodelling phase, TGF-β1 levels were significantly elevated in Groups 3, 4, and 5, which coincided with reduced connective-tissue thickness. In the L/MEL group, increased connective-tissue thickness represented oedematous and structurally disorganised granulation tissue rather than productive collagen deposition. The fragmented collagen pattern and high caspase-3 activity suggest ongoing matrix turnover and impaired progression toward maturation. Thus, the thickened wound bed in the meloxicam group reflects delayed and disorganised repair rather than improved structural healing. The biological response to TGF-β1 depends on cell type; it stimulates proliferation of mesenchymal cells (fibroblasts) while inhibiting epithelial, endothelial, neural, and haematopoietic cells during the G1/S cell-cycle transition [12]. Its signalling interacts with several other growth-factor pathways. Our findings support these mechanisms, as Groups 4 and 5 showed reduced re-epithelialisation zone length and wound-border distance during proliferation and remodelling, accompanied by decreased connective-tissue thickness in the remodelling phase. The effects of local anaesthetics on wound healing depend on dose, administration route, and dosing interval. These agents influence inflammatory and proliferative phases by reducing cytokine and chemokine release, suppressing inflammation, and decreasing collagen synthesis [27]. Levobupivacaine has negative effects on early healing by impairing collagen formation during the first eight days, after which its effects become beneficial [6], consistent with our findings regarding wound depth and connective-tissue thickness. Adjuvants such as opioids and NSAIDs appear to potentiate these effects.

Opioid receptors are expressed on keratinocytes, fibroblasts, and neurons, and appropriate analgesia enhances healing. Endogenous opioids released by inflammatory cells may induce ischaemic wound healing, whereas exogenous opioids promote granulation, collagen deposition, epidermal and dermal organisation, and angiogenesis. In contrast, NSAIDs exert antiproliferative effects on vasculature and skin and may delay healing [27]. Our results regarding vessel number and area support these observations. Although meloxicam increased the number of CD31-positive vessels in the early phase of healing, these vessels were structurally immature, with consistently narrow lumina and pronounced regression in the remodelling phase. This pattern reflects COX-2 inhibition–related impairment of endothelial maturation and represents dysregulated angiogenesis rather than effective proliferative healing. In contrast, buprenorphine was associated with wider-lumen, more mature vessels, suggesting a more stable and functionally favourable angiogenic response. Thus, while both treatments increased vessel counts, the quality and maturation of the newly formed vessels differed substantially, explaining why meloxicam demonstrates overall antiproliferative effects despite stimulating early vessel sprouting. However, NSAID treatment did not significantly affect vessel perimeter in the initial stage. Levobupivacaine and catheter placement caused even greater disturbances in the vessel perimeter. By day 7 following L/MEL application, no intergroup differences in vascular parameters remained.

Opioid receptors belong to the G-protein family [33] and are upregulated in injured tissue. Opioid agonists binding to these receptors accelerate healing. Wang et al. [27] reported that δ- and κ-receptors do not influence early healing, whereas morphine promotes wound closure after day 8. μ-opioid receptors regulate neuropeptide expression, supporting revascularisation and granulation during the proliferative phase [27].

Revascularisation is essential for wound healing, providing oxygen and nutrients to newly formed granulation tissue. Endothelial cells maintain homeostasis by releasing angiocrine factors and regulating the permeability of proteins, ions, and other key molecules [34]. We observed numerous vessels during inflammation in Groups 4 and 5, and during proliferation in Group 4. However, vessels in Group 4 had narrow lumina in both phases.

The vascular phenotype observed in the levobupivacaine/meloxicam group can be interpreted through the known biology of COX-2 inhibition. Meloxicam suppresses COX-2–dependent PGE_2_ production [4], and PGE_2_ is recognised as an important permissive signal for endothelial proliferation and maturation during wound angiogenesis [8,34]. When PGE_2_ levels fall, VEGF-driven angiogenesis becomes unbalanced, favouring excessive early sprouting without adequate maturation of endothelial junctions. This results in a high number of structurally immature capillaries with very narrow lumina, essentially a chaotic sprouting pattern, which is consistent with the marked vascular regression we observed at later stages. PECAM-1 (CD31) is expressed on nearly all vascular cells except erythroid cells and is abundant at endothelial junctions [17,35,36]. Inflammation prompts endothelial retraction, disrupting junctions and enabling extravasation [17,18]. CD31-mediated endothelial protection was evident in Groups 1 (days 3, 10, 21) and 2 (days 3 and 10).

Apoptosis involves cysteine proteases, with caspase-3 acting as a major executioner enzyme [15,37]. It proceeds asynchronously and without inflammation. Apoptosis and autophagy are interconnected self-regulated processes [38].

This study has several limitations. One important methodological constraint is the use of serial punch biopsies along the same 4 cm incision. Although each biopsy removed only a 6 mm segment of tissue, the samples were deliberately collected in a strictly cranial-to-caudal order, ensuring that the day 3, day 10, and day 21 biopsies originated from spatially distinct, non-overlapping regions of the wound. Given the total wound length, each sampling site was separated by approximately 6–8 mm of intact tissue, which minimised direct mechanical overlap.

However, we acknowledge that the surgical wound behaves as a continuous biological compartment. Thus, even when anatomically separated, later biopsy zones may still have been affected by local inflammatory and reparative spillover resulting from earlier sampling. As a consequence, the day-21 CD31 and caspase-3 profiles may partially reflect tissue responses to the day-10 biopsy, rather than late-stage remodelling alone.

CRP is not the most reliable APP in rats. Secondly, key TGF-β activators such as plasmin and matrix metalloproteinases were not analysed. Thirdly, the measurement of vessel perimeter may be affected by the angle of microtome cutting.

Clinical relevance: Following major surgical procedures, multimodal analgesia is optimal. Our findings suggest that levobupivacaine/buprenorphine administered via wound catheter has minimal adverse effects on healing due to favourable angiogenesis and moderate caspase-3 activity across all four stages of wound healing.

## 5. Conclusions

In conclusion, the highest TGF-β1 concentrations were observed in the levobupivacaine/meloxicam group at all time points, whereas levobupivacaine alone did not significantly alter TGF-β1 levels over time. NSAID treatment exerted the greatest influence on average vessel perimeter and vessel-area percentage, while opioids had no such effect. Levobupivacaine alone induced local catabolic activity, which became even more pronounced when combined with meloxicam. Meloxicam also triggered abundant angiogenesis with narrow-lumen vessels and markedly increased caspase-3 expression. In contrast, buprenorphine supported angiogenesis with vessels of moderate lumen size, suppressed caspase-3 activity during the inflammatory phase, and was associated with a gradual increase in TGF-β1 expression.

Both meloxicam and buprenorphine attenuated the inflammatory response; however, meloxicam additionally induced oxidative stress, impaired mitochondrial activity, and delayed caspase activation. These findings suggest that levobupivacaine/buprenorphine administered via a wound catheter may be the most favourable option, exerting minimal negative effects on wound healing through balanced angiogenesis and moderate apoptotic activity.

Future studies should focus on identifying optimal long-term local or regional analgesia protocols that minimise interference with wound healing, including determining appropriate dosing strategies and intervals of administration.

## Figures and Tables

**Table 1:** Comparison of TGF, CRP, and WBC levels within five groups at four time points during the wound healing process.

	Day	0	3	10	21	*p*-Value **
Group						
TGFß1 (pg/mL)						
1		91.88 (8.86)	62.90 (18.58)	98.03 (8.42)	79.63 (13.44)	0.003 ^d0–d3;d3–d10^
2		91.88 (8.86)	70.14 (12.06)	86.87 (14.88)	96.14 (6.92)	0.010 ^d0–d3;d3–d21^
3		91.88 (8.86)	85.91 (8.82)	102.35 (16.29)	102.39 (10.09)	0.093
4		91.88 (8.86)	92.91 (15.64)	104.16 (14.53)	124.08 (4.85)	0.002 ^d0–d21;d3–d21^
5		91.88 (8.86)	84.40 (14.79)	96.83 (18.88)	116.03 (20.37)	0.043 ^d3–d21^
*p*-value *		1.000	0.021 ^G1–G4^	0.426	<0.001 ^G1–G4;G1–G5;G2–G4^	0.016 ***
CRP (ng/mL)						
1		398.10 (46.25)	509.51 (56.02)	570.65 (55.58)	585.38 (85.29)	0.001 ^d0–d10;d0–d21^
2		398.10 (46.25)	568.03 (84.67)	707.82 (208.52)	498.68 (206.97)	0.039 ^d0-d10^
3		398.10 (46.25)	633.41 (119.76)	547.19 (67.98)	631.29 (267.31)	0.085
4		398.10 (46.25)	674.77 (92.09)	575.62 (45.09)	600.27 (119.52)	0.001 ^d0–d3;d0–d10;d0–d21^
5		398.10 (46.25)	706.27 (86.13)	578.75 (48.28)	544.11 (101.12)	<0.001 ^d0–d3;d0–d10;d0–d21;d3–d21^
*p*-value *		1.000	0.016 ^G1–G5^	0.163	0.767	0.124 ***
WBC (×10^9^/L)						
1		7.50 (2.03)	9.52 (2.07)	8.43 (1.94)	10.28 (1.37)	0.085
2		8.77 (1.15)	8.85 (1.41)	8.82 (1.95)	9.83 (0.94)	0.517
3		8.15 (2.80)	9.17 (2.47)	6.10 (2.66)	8.75 (2.61)	0.222
4		8.51 (2.08)	10.40 (1.44)	8.60 (0.90)	5.37 (3.43)	0.013 ^d3–d21^
5		8.15 (1.09)	9.40 (2.84)	7.75 (2.08)	5.58 (1.70)	0.030 ^d3–d21^
*p*-value *		0.895	0.773	0.152	0.001 ^G1–G4;G1–G5;G2–G4;G2–G5^	0.004 ***

Abbreviations: d0–d21 indicate days (day 0 to day 21); G1–G5 indicate experimental groups. * *p*-value obtained for differences between five tested groups; for each time point separately (column-wise); results of one-way ANOVA with labelled significant pairwise associations after Tukey’s HSD post hoc tests with Benjamini–Hochberg *p*-value adjustment. ** *p*-value obtained for differences between four time points; for each tested group separately (row-wise); results of one-way ANOVA with labelled significant pairwise associations after Tukey’s HSD post hoc tests with Benjamini–Hochberg *p*-value adjustment. *** Overall *p*-value obtained for differences when both groups and time points are taken into account; results of two-way ANOVA.

**Table 2:** Comparison of length of re-epithelization zone (L), thickness of the connective tissue (T), number of vessels and their average perimeter, and number of caspase3-positive cells levels within five groups, at four time points during wound healing process.

	Day	0	3	10	21	*p*-Value **
Group						
L (µm)						
1		1018.08 (348.09)	1484.71 (311.3)	2686.35 (669.61)	1279.38 (293.76)	<0.001 ^d0–d10;d3–d10;d10–d21^
2		1018.08 (348.09)	2942.68 (2179.85)	2604.68 (570.81)	1186.53 (220.08)	0.053
3		1018.08 (348.09)	2109.38 (404.83)	2479.08 (799.25)	1206.72 (187.17)	0.001 ^d0–d3;d0–d10;d3–d21;d10–d21^
4		1018.08 (348.09)	2927.92 (741.52)	2117.97 (870.32)	1113.33 (263.34)	0.002 ^d0–d3;d3–d21^
5		1018.08 (348.09)	2623.74 (1203.19)	1738.42 (437.64)	1058.80 (329.89)	0.009 ^d0–d3;d3–d21^
*p*-value *		1.000	0.389	0.192	0.655	0.295 ***
T (µm)						
1		948.89 (605.50)	1631.47 (561.37)	1735.77 (636.93)	1947.45 (686.64)	0.216
2		948.89 (605.50)	1168.93 (474.20)	1762.30 (446.29)	1885.12 (559.45)	0.033 ^none^
3		948.89 (605.50)	1146.64 (436.9)	2419.91 (393.40)	1782.40 (156.14)	<0.001 ^d0–d10;d3–d10;d0–d21^
4		948.89 (605.50)	1062.81 (497.52)	1842.71 (371.32)	1694.24 (329.92)	0.012 ^d0–d10;d3–d10^
5		948.89 (605.50)	868.54 (411.06)	1777.93 (821.38)	1679.39 (500.09)	0.047
*p*-value *		1.000	0.206	0.194	0.839	0.662 ***
Number of vessels						
1		26.33 (17.04)	36.00 (11.14)	84.50 (55.69)	66.33 (23.24)	0.134
2		26.33 (17.04)	32.33 (6.59)	47.33 (19.78)	34.01 (16.78)	0.233
3		26.33 (17.04)	29.25 (7.37)	64.50 (9.2)	43.83 (16.55)	0.002 ^d0–d10;d3–d10^
4		26.33 (17.04)	57.00 (29.48)	91.33 (26.9)	33.67 (8.12)	0.001 ^d0–d10;d10–d21^
5		26.33 (17.04)	49.83 (29.74)	69.83 (41.89)	44.05 (15.59)	0.225
*p*-value *		1.000	0.210	0.237	0.016 ^G1–G2;G1–G4^	0.451 ***
Average perimeter of vessels						
1		619.62 (565.85)	434.95 (535.37)	7953.87 (16,460.01)	1496.57 (667.16)	0.583
2		619.62 (565.85)	255.55 (200.49)	1307.02 (887.60)	652.48 (386.8)	0.038 ^d3–d10^
3		619.62 (565.85)	240.79 (273.13)	1294.16 (388.12)	867.07 (464.98)	0.011 ^d3–d10^
4		619.62 (565.85)	303.67 (326.71)	1758.17 (472.50)	699.19 (229.4)	<0.001 ^d0–d10;d3–d10;d10–d21^
5		619.62 (565.85)	940.75 (1020.22)	1438.29 (556.29)	1014.9 (347.55)	0.380
*p*-value *		1.000	0.250	0.459	0.021 ^G1–G2;G1–G4^	

Abbreviations: d0–d21 indicate days (day 0 to day 21); G1–G5 indicate experimental groups. * *p*-value obtained for differences between five tested groups; for each time point separately (column-wise); results of one-way ANOVA with labelled significant pairwise associations after Tukey’s HSD post hoc tests with Benjamini–Hochberg *p*-value adjustment. ** *p*-value obtained for differences between four time points; for each tested group separately (row-wise); results of one-way ANOVA with labelled significant pairwise associations after Tukey’s HSD post hoc tests with Benjamini–Hochberg *p*-value adjustment. *** Overall *p*-value obtained for differences when both groups and time points are taken into account; results of two-way ANOVA. Numbers in cells represented as mean with standard deviation in brackets. Bold *p*-values indicate statistically significant differences (*p* < 0.05).

## Data Availability

The original contributions presented in this study are included in the article/Supplementary Material. Further inquiries can be directed to the corresponding author.

## Supplementary Materials

The following supporting information can be downloaded at: https://www.mdpi.com/article/10.3390/vetsci12121214/s1, Figure S1. Boxplots of TGF-β1, vessel number, vessel area percentage, and caspase-3; Figure S2. Correlation between leukocytes and caspase-3; Figure S3. Correlation between CRP and albumin; Figure S4. Correlation between CRP and leukocytes; Figure S5. Correlation between total vessel area and average vessel area; Figure S6. Correlation between number of vessels and average vessel area; Figure S7. Correlation between number of vessels and caspase-3; Table S1: Comparison of red blood cell and albumin levels between five groups and four time points during healing process; Table S2: Score results of the following data: Distance between borders (S), depth of the wound from the epidermis (D) and thickness of the natural dermis (N) in four time points and five groups during wound healing process; Table S3: Comparison between groups of total wound area, total vessels area, average vessels area, percentage vessels area/ total area, and average total caspase3-positive levels cell during inflammatory, proliferative, and remodelling process of wound healing. Supporting data and materials are provided in the Supplementary Materials.

## Abbreviations

The following abbreviations are used in this manuscript:

ANOVA	Analysis of variance
APP	Acute phase protein
BUP	Buprenorphine
CD31/PECAM-1	Platelet/endothelial cell adhesion molecule-1
COX-2	Cyclooxygenase-2
CRP	C-reactive protein
D	Wound depth
ECM	Extracellular matrix
ELISA	Enzyme-linked immunosorbent assay
GABA	Gamma-aminobutyric acid
HSD	Tukey’s honestly significant difference test
IHC	Immunohistochemistry
IL-6	Interleukin-6
L	Levobupivacaine
Lr	Length of the re-epithelialisation zone
MEL	Meloxicam
N	Natural dermis thickness
NPP	Negative phase protein
NSAID	Nonsteroidal anti-inflammatory drug
RBC	Red blood cell
S	Distance between wound borders
SD	Standard deviation
T	Connective-tissue thickness
TGF-β1	Transforming growth factor beta-1
WBC	White blood cell
