# Supplementary material for: Role of Cytokines in Wound Healing Following Wound Catheter Analgesia in Rats
Source: Vet Sci. 2025 Dec 18;12(12):1214. doi: 10.3390/vetsci12121214 (PMC12737617; doi:10.3390/vetsci12121214)
Supplement: Supplementary file 1 [file vetsci-12-01214-s001.zip › vetsci-4024390-supplementary.pdf]

Figure S1. Boxplots of TGF-β1, vessel number, vessel area percentage and caspase-3.

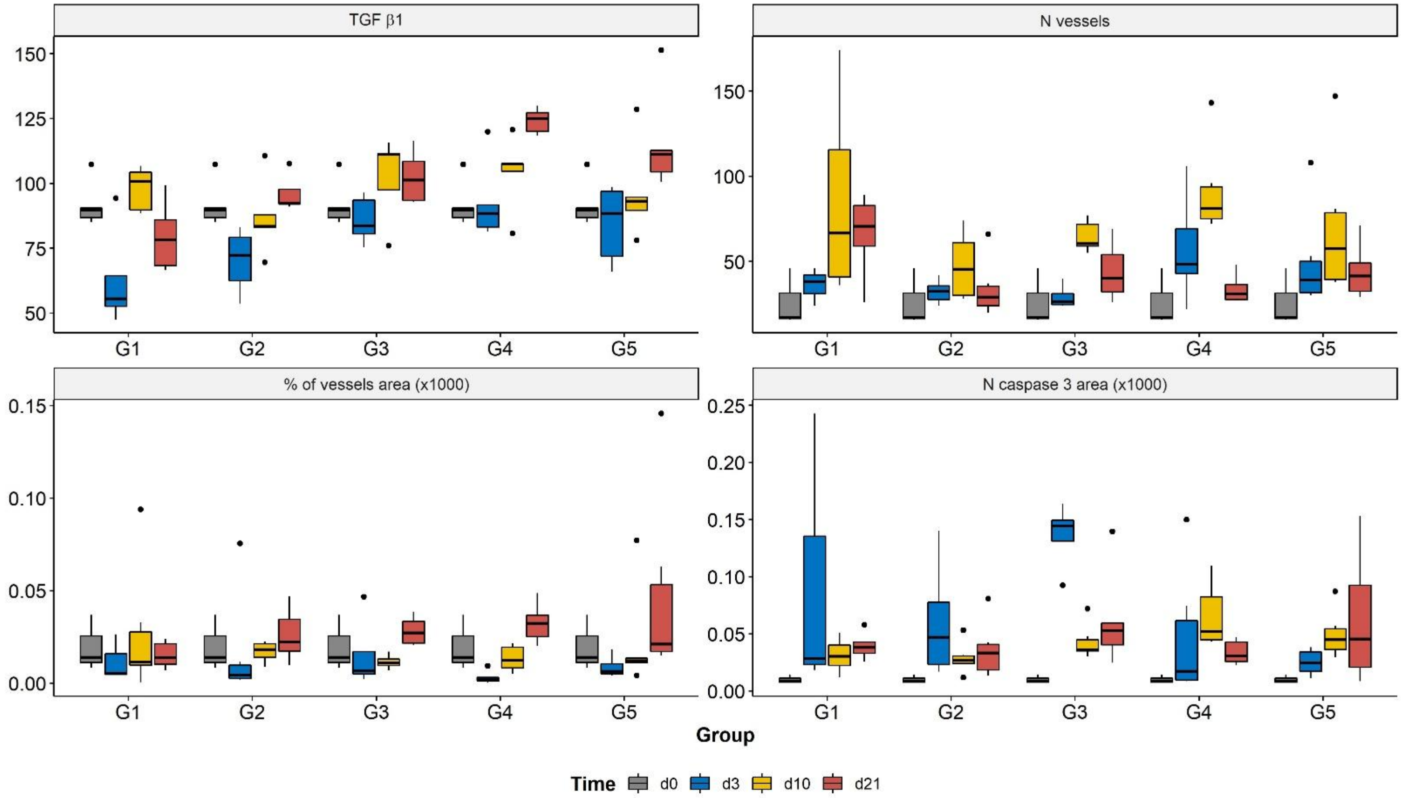

Figure S2. Correlation between leukocytes and caspase-3.

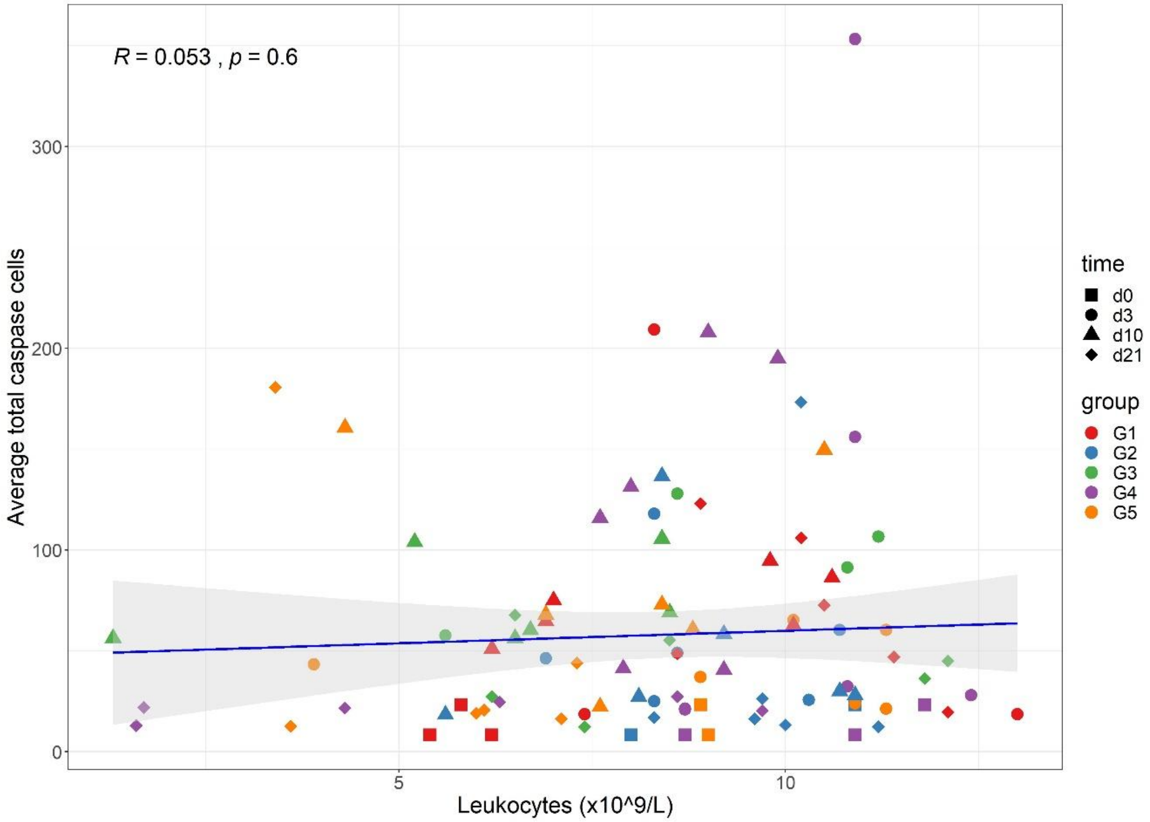

Figure S3. Correlation between CRP and albumin.

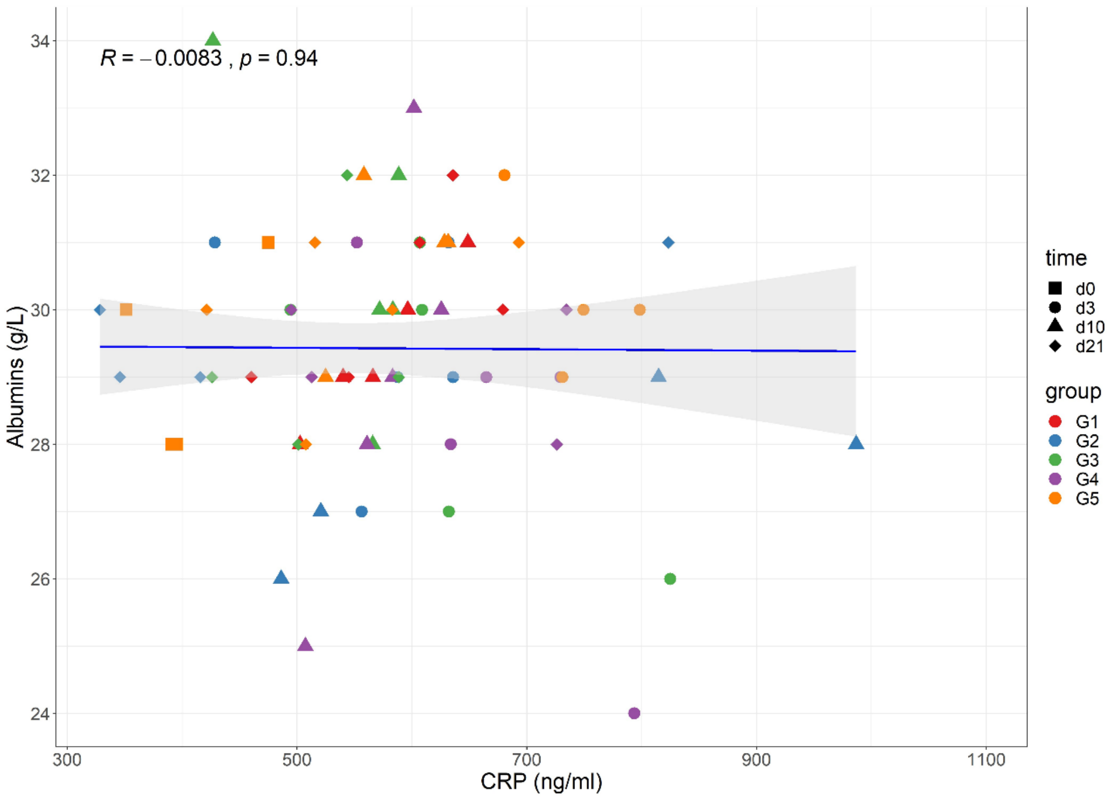

Figure S4. Correlation between CRP and leukocytes.

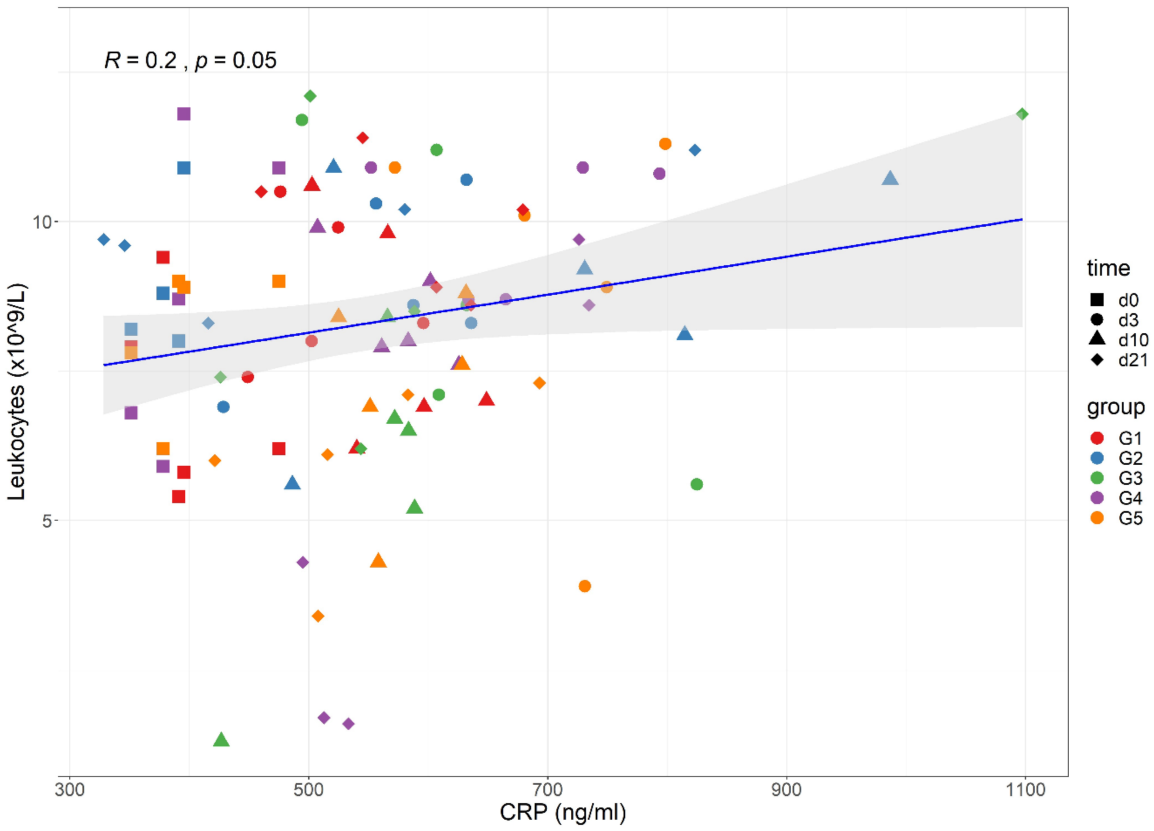

Figure S5. Correlation between total vessel area and average vessel area.

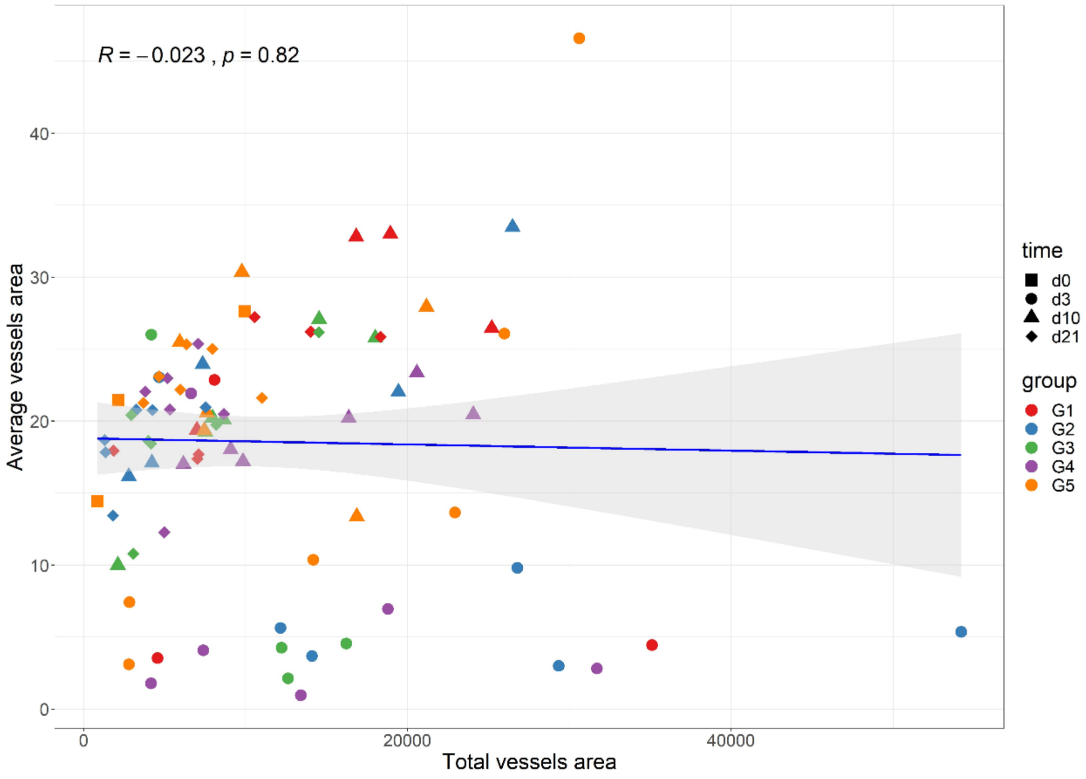

Figure S6. Correlation between number of vessels and average vessel area.

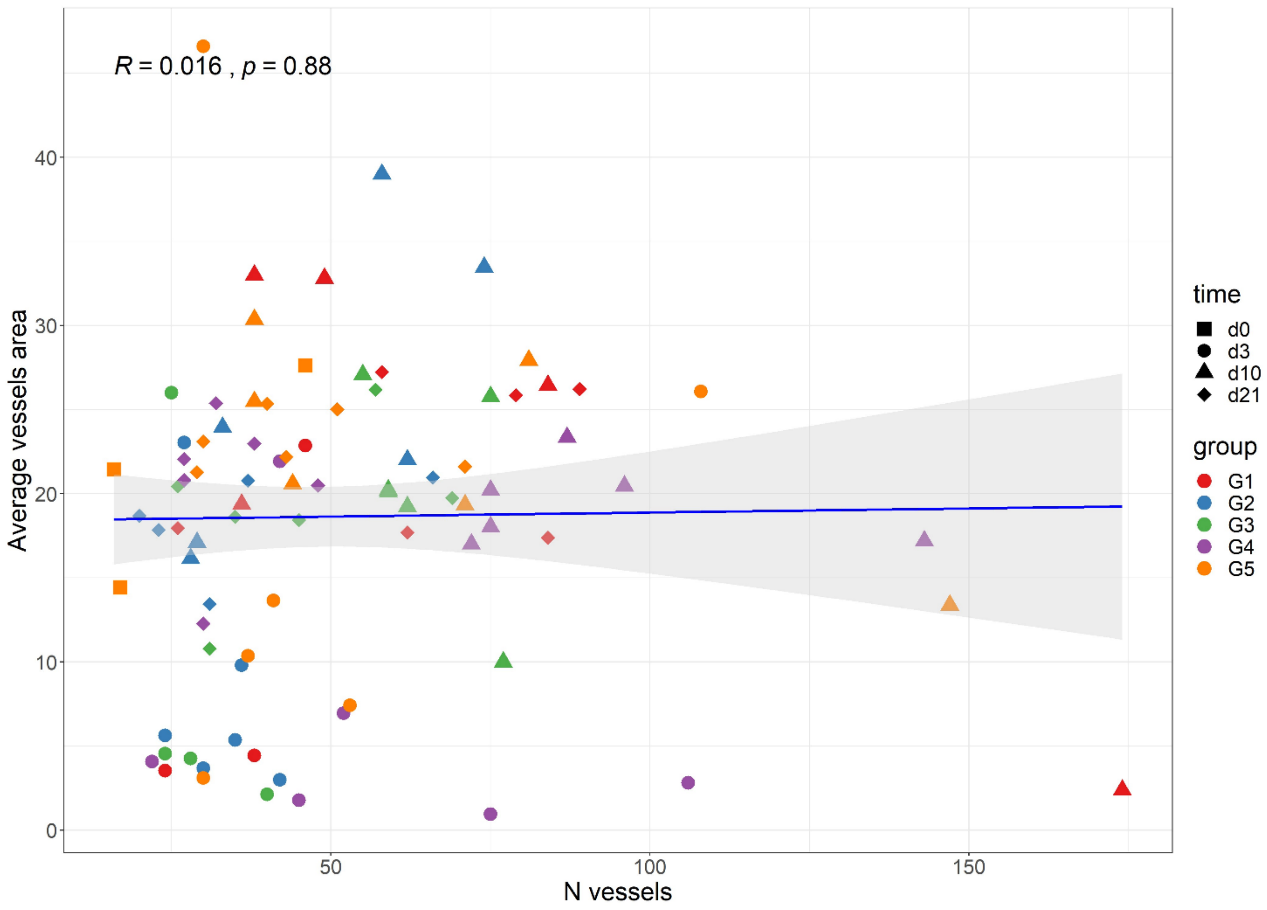

Figure S7. Correlation between number of vessels and caspase-3

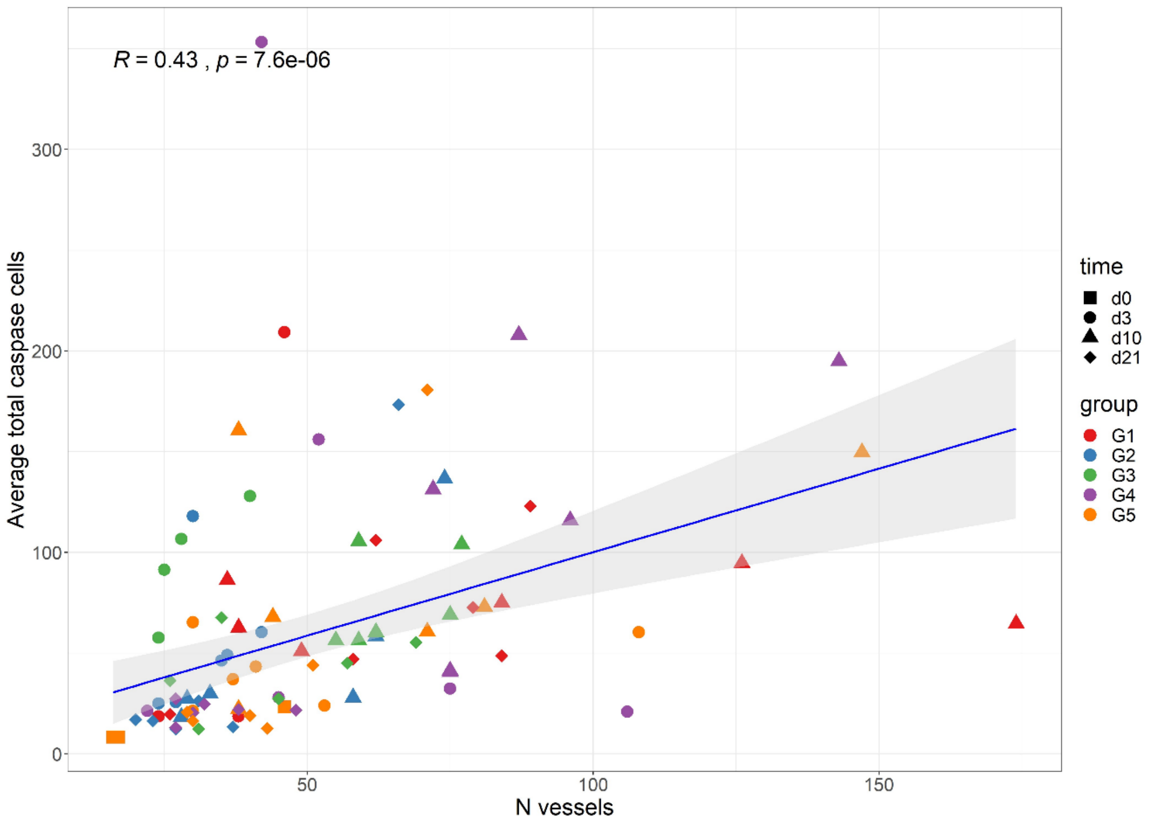

Table S1. Comparison of red blood cell and albumin levels between 5 groups and four time points during healing process.

**Day**<br>**Group**	**0**	**3**	**10**	**21**	**P-value****
**RBC (x 10^{12}/L)**					
**1**	6.57 (0.59)	5.50 (0.61)	6.08 (0.58)	7.36 (0.71)	**<0.001**$^{\text{d0-d3;d3-d21;d10-d21}}$
**2**	6.24 (0.95)	4.81 (0.72)	5.61 (1.30)	7.22 (0.79)	**0.003**$^{\text{d3-d21;d10-d21}}$
**3**	6.81 (1.10)	5.12 (0.45)	5.22 (1.11)	7.62 (0.45)	**<0.001**$^{\text{d0-d3;d0-d10;d3-d21;d10-d21}}$
**4**	6.81 (1.10)	4.74 (0.31)	5.94 (1.01)	6.91 (2.29)	**0.045**^{none}
**5**	6.29 (0.99)	4.77 (0.89)	5.85 (1.63)	7.92 (1.27)	**0.003**$^{\text{d3-d21;d10-d21}}$
**P-value***	0.745	0.205	0.741	0.704	0.796***
**Albumin (g/L)**					
**1**	29.25 (1.50)	/	29.40 (1.14)	30.20 (1.30)	0.507
**2**	29.25 (1.50)	29.40 (1.67)	27.51 (1.29)	29.75 (0.96)	0.153
**3**	29.25 (1.50)	28.80 (2.17)	30.81 (2.28)	29.50 (1.73)	0.456
**4**	29.25 (1.50)	28.21 (2.59)	29.05 (2.92)	29.25 (0.96)	0.873
**5**	29.25 (1.50)	30.25 (1.26)	30.75 (1.26)	30.06 (1.22)	0.461
**P-value***	1.000	0.497	0.128	0.807	0.533

* P-value obtained for differences between five tested groups

** P-value obtained for differences between four time points

*** P-value obtained for differences when both groups and time points are taken into account

**5**	619.62 (565.85)	940.75 (1020.22)	1438.29 (556.29)	1014.90 (347.55)	0.380
**P-value***	1.000	0.250	0.459	**0.021**$^{G1-G2;G1-G4}$	0.828***
**No cas3+ cells area (x 10^{6})**					
**1**	9.9 (3.9)	96.4 (127.0)	31.1 (14.3)	39.2 (11.2)	0.203
**2**	9.9 (3.9)	59.0 (47.3)	28.9 (13.6)	36.2 (24.8)	0.145
**3**	9.9 (3.9)	136 (30.6)	42.8 (15.5)	61.2 (40.6)	**<0.001**$^{d0-d3;d3-d10;d3-d21}$
**4**	9.9 (3.9)	46 (56.8)	65.3 (28.2)	33.6 (10.6)	0.172
**5**	9.9 (3.9)	25.1 (10.9)	49.5 (20.7)	62.2 (56.3)	0.114
**P-value***	1.000	0.054	**0.02**$^{G1-G4;G2-G4}$	0.404	**0.012*** **

* P-value obtained for differences between five tested groups
** P-value obtained for differences between four time points
*** P-value obtained for differences when both groups and time points are taken into account

Table S2. Score results of the following data: Distance between borders (S), depth of the wound from the epidermis (D) and thickness of the natural dermis (N) in four time points and five groups during wound healing process.

**Day / Group**	**0**	**3**	**10**	**21**	**P-value****
**S (μm)**					
**1**	487.55 (244.01)	759.66 (346.10)	1613.92 (862.02)	1058.32 (278.41)	**0.044**$^{d0-d10}$
**2**	487.55 (244.01)	1695.71 (1091.30)	1518.53 (546.95)	2049.71 (2967.62)	0.655
**3**	487.55 (244.01)	1280.33 (247.30)	1973.63 (671.94)	991.19 (210.70)	**0.001**$^{d0-d10;d10-d21}$
**4**	487.55 (244.01)	2193.94 (697.32)	1326.56 (316.16)	942.9 (196.36)	**<0.001**$^{d0-d3;d3-d10;d3-d21}$
**5**	487.55 (244.01)	1690.76 (866.58)	1377.49 (393.63)	860.21 (233.35)	**0.022**$^{d0-d3}$
**P-value***	1.000	0.102	0.366	0.559	0.478***
**D (μm)**					
**1**	244.52 (91.44)	244.4 (65.88)	401.05 (215.70)	220.78 (70.61)	0.185
**2**	244.52 (91.44)	755.48 (780.75)	395.13 (206.04)	183.36 (69.83)	0.168
**3**	244.52 (91.44)	441.91 (102.89)	385.1 (178.85)	228.1 (75.81)	**0.038**^{none}
**4**	244.52 (91.44)	511.6 (196.62)	506.98 (443.85)	165.33 (81.75)	0.117

Group \ Day	**0**	**3**	**10**	**21**	**P-value****
**5**	244.52 (91.44)	919.16 (595.37)	288.07 (40.97)	212.46 (129.06)	**0.009**$^{d3-d10;d3-d21}$
**P-value***	1.000	0.311	0.741	0.704	0.317***
**N (μm)**					
**1**	1446.97 (624.42)	2181.68 (757.09)	1410.86 (594.9)	2520.20 (572.46)	**0.038**$^{d10-d21}$
**2**	1446.97 (624.42)	2045.91 (363.63)	1261.67 (417.37)	2148.09 (540.15)	**0.014**$^{d3-d10;d10-d21}$
**3**	1446.97 (624.42)	1837.33 (127.47)	1425.87 (417.11)	2146.96 (466.44)	**0.037**$^{d10-d21}$
**4**	1446.97 (624.42)	1619.06 (541.51)	1275.24 (582.8)	1899.27 (350.87)	0.244
**5**	1446.97 (624.42)	1773.58 (536.04)	2025.37 (937.82)	1906.60 (517.30)	0.677
**P-value***	1.000	0.438	0.261	0.215	0.562***4

* P-value obtained for differences between five tested groups

** P-value obtained for differences between four time points

*** P-value obtained for differences when both groups and time points are taken into account

Table S3. Comparison between groups of total wound area, total vessels area, average vessels area, percentage vessels area/ total area and average total caspase3 positive cell during inflammatory, proliferative and remodelling process of wound healing.

Day / Group	0	3	10	21	P-value**
**total wound area**					
**1**	1621859.8 (1432273.14)	850186.21 (178439.2)	2862364.04 (1489429.61)	1767600.21 (1058072.49)	0.154
**2**	1621859.8 (1432273.14)	1292692.77 (858924.21)	1673520.65 (868573.51)	953785.34 (628571.88)	0.533
**3**	1621859.8 (1432273.14)	701876.85 (143840.76)	1789062.05 (268447.91)	719588.04 (289369.89)	**0.013**$^{d3-d10;d10-d21}$
**4**	1621859.8 (1432273.14)	2285835.57 (834725.2)	1910763.57 (1088284.61)	660183.64 (135688.32)	**0.035**$^{d3-d21}$
**5**	1621859.8 (1432273.14)	2179683 (1745103.18)	1880308.96 (936214.74)	949486.01 (570856.75)	0.374
**P-value***	1.000	0.095	0.282	**0.036**$^{G1-G4}$	0.306***
**total vessels area**					
**1**	4312.41 (4911.28)	15928.72 (16716.74)	126826.28 (233872.79)	9815.43 (5824.15)	0.451
**2**	4312.41 (4911.28)	23550.23 (17662.19)	161758.63 (366813.98)	3249.88 (2406.2)	0.498
**3**	4312.41 (4911.28)	11318.29 (5085.39)	9795.68 (5641.32)	6148.36 (4534.77)	0.230
**4**	4312.41 (4911.28)	13687.72 (10308.07)	14346.7 (7106.92)	5834.55 (1734.51)	0.083
**5**	4312.41 (4911.28)	16561.18 (11920.57)	11463.15 (6135.25)	6601.87 (2609.94)	0.095
**P-value***	1.000	0.627	0.502	0.081	0.820***
**average vessels area**					
**1**	21.16 (6.6)	10.28 (10.9)	73.93 (125.76)	22.06 (4.82)	0.552
**2**	21.16 (6.6)	8.42 (7.55)	25.28 (9.14)	18.75 (2.9)	**0.005**$^{d3-d10}$
**3**	21.16 (6.6)	9.23 (11.23)	20.4 (6.05)	19.04 (4.94)	0.104
**4**	21.16 (6.6)	6.42 (7.88)	19.38 (2.44)	20.67 (4.47)	**0.001**$^{d0-d3;d3-d10;d3-d21}$
**5**	21.16 (6.6)	17.87 (16.09)	22.85 (6.27)	23.09 (1.74)	0.775
**P-value***	1.000	0.474	0.415	0.283	0.741***
**percentage vessels area /total area**					
**1**	19.8 (15.1)	12.1 (12.5)	26.6 (34.7)	15.3 (7.1)	0.770
**2**	19.8 (15.1)	16.6 (29.1)	17.1 (5.34)	25.9 (13.9)	0.812
**3**	19.8 (15.1)	15.5 (20.8)	11.4 (3.55)	28.1 (7.68)	0.144
**4**	19.8 (15.1)	2.95 (3.28)	13.3 (6.99)	32.4 (10.3)	**<0.001**$^{d3-d21;d10-d21}$
**5**	19.8 (15.1)	8.65 (5.46)	21.7 (27.4)	47.2 (51.6)	0.249

Day / Group	0	3	10	21	P-value**
**P-value***	1.000	0.687	0.693	0.293	0.675***
**average total cas3+ cells**					
**1**	13.33 (8.66)	82.22 (110.08)	72.39 (16.18)	69.5 (39.06)	0.313
**2**	13.33 (8.66)	54.06 (34.25)	49.78 (44.68)	43.11 (63.99)	0.646
**3**	13.33 (8.66)	95.92 (29.6)	75.28 (23.36)	40.67 (19.81)	**0.001**$^{d0-d3;d0-d10;d3-d21}$
**4**	13.33 (8.66)	102 (133.78)	122.06 (72.07)	21.5 (4.86)	0.119
**5**	13.33 (8.66)	41.89 (18.21)	89.06 (54.33)	48.89 (65.5)	0.154
**P-value***	1.000	0.653	0.135	0.499	0.596***

* P-value obtained for differences between five tested groups

** P-value obtained for differences between four time points

*** P-value obtained for differences when both groups and time points are taken into account
